# Mortality is not associated with paclitaxel-coated devices usage in peripheral arterial disease of lower extremities

**DOI:** 10.1038/s41598-021-97675-9

**Published:** 2021-09-14

**Authors:** Dai Sik Ko, Gi Hwan Bae, Sang Tae Choi, Jaehun Jung, Jin Mo Kang

**Affiliations:** 1 Division of Vascular Surgery, Department of Surgery, Gachon University Gil Medical Center, 34 Namdong-daero 774beon-gil, Namdong-gu, Incheon, 21565 Republic of Korea; 2 Artificial Intelligence and Big-Data Convergence Center, Gachon University Gil Medical Center, 38-13 Dokjeom, Incheon, 21565 Republic of Korea; 3 Department of Preventive Medicine, Gachon University College of Medicine, Incheon, Republic of Korea

**Keywords:** Vascular diseases, Medical research

## Abstract

A recent meta-analysis addressed increased risk of death following revascularization with paclitaxel-coated devices in femopopliteal artery. We evaluated differences in all-cause mortality and amputation free survival between peripheral arterial disease (PAD) patients who were treated with paclitaxel-coated devices and non-paclitaxel-coated devices. This was retrospective population-based cohort study from the National Health Insurance Service claims in South Korea from 2015 to 2019. Multivariate Cox regression analyses after propensity score matching were applied to identify all-cause mortality and amputation-free survival. After propensity score matching, there were 6090 patients per group. The median follow-up days was 580 days (interquartile range [IQR] 240–991 days) and 433 days (IQR 175–757 days) for the non-paclitaxel-coated device group and paclitaxel-coated device group, respectively. Multivariate analysis adjusted for age, sex, diabetes, hypertension, warfarin, and new oral anticoagulants showed that the mortality rate associated with paclitaxel-coated devices was not significantly higher than non-paclitaxel-coated devices (hazard ratio [HR] 0.992; 95% CI 0.91–1.08). The rate of amputation events was higher in patients with paclitaxel-coated devices than those with non-paclitaxel-coated devices (HR 1.614; 95% CI 1.46–1.78). In this analysis, the mortality rate in patients with PAD was not associated with the use of paclitaxel-coated devices, despite a higher amputation rate.

## Introduction

Peripheral arterial disease (PAD) has become a worldwide health problem affecting more than 118 million patients globally as of 2017^1^. Patients with PAD have a three to five times higher mortality rate than patients without PAD^2^. Endovascular intervention has gained global acceptance as the primary approach of revascularization for patients with PAD; however, post-procedural restenosis is common. From this perspective, paclitaxel-coated devices have become increasingly popular in the treatment of PAD in reducing the high restenosis rate^3,4^. Practice guidelines from societies in the USA and Europe as well as the 2018 SCAI consensus guidelines included drug-eluting balloons (DEBs) and drug-eluting stents (DES) as treatment options to lower the re-intervention rate^5–7^.

A potential long-term safety concern was raised during a recent meta-analysis by Katsanos et al.They identified an increased mortality rate associated with the use of paclitaxel devices for femoropopliteal PAD^8^. One of the major limitations of their study was that patients lost to follow-up could not be appropriately accounted for. Moreover, the cause of death was not available for most studies included in the study by Katsanos et al. The Food and Drug Administration requested researchers to collect long-term safety (including mortality) and effectiveness data to draw definitive conclusions^9^. Accordingly, some publications showed opposite findings based on individual patient-level data and various forms of adjusted comparisons^10–13^. However, there are limitations to using data from a single industry-sponsored study and limited follow-up duration.

The National Health Insurance Service (NHIS) in Korea delivers a government-controlled single-payer obligatory insurance plan covering almost the entire Korean population^14^. The NHIS pays costs based on the billing records of health care providers. To carry out these processes, the NHIS built a data warehouse to collect the required information on medical treatments and insurance eligibility, which are income-based insurance contributions, demographic variables, date of death, records on inpatient and outpatient usage, and prescription records. A real-world clinical data could be a practical alternative for all-cause mortality analysis.

This study aimed to address concerns related to long-term survival after treating the lower extremities with paclitaxel-coated devices using a propensity score-matched retrospective analysis of national health insurance claims from South Korea.

## Methods

The Institutional Review Board of the Gachon University Gil Medical Center (Incheon, South of Korea) reviewed and approved the study protocol in compliance with governmental laws and regulations (protocol GFIRB2019-200), and the requirement for informed consent was waived as we only accessed deidentified, previously collected data. All study methods were carried out based on the Declaration of Helsinki.

### Study design

This population-based retrospective cohort study aimed to evaluate the effects of paclitaxel-coated devices on all-cause mortality in patients with PAD. Customized data of patients who were diagnosed with PAD and had been treated with interventional modalities from January 1, 2015, to December 31, 2018, were extracted from the NHIS database, and patients were followed up until December 31, 2019. The diagnoses were coded according to the *Korean Standard Classification of Diseases Version 7*, which is based on the *International Classification of Diseases, Tenth Revision* (*ICD-10*)^15^.

Patients with PAD undergoing interventional treatments were defined as those who met all the following two criteria: (1) had ICD-10 codes corresponding to atherosclerosis of native arteries of the extremities (I702); (2) had procedure codes of percutaneous balloon angioplasty of arteries of the extremities (M6597) and percutaneous stent placement of arteries of the extremities (M6605); and (3) had device codes related to four interventional modalities, i.e., plain balloon angioplasty (PBA), bare metal stent (BMS), DEB, and DES. Their specific codes are provided in detail in Supplementary Table S1. Patients without eligibility data who underwent amputation of the lower extremities before the indexing procedure were excluded. The usage of drug-coated devices was first claimed on the NHIS in September 2015; therefore, only data after September 2015 were included.

### Measurements

Interventional modalities (PBA, BMS, DEB, and DES) were defined as at least one procedure code being recorded in the claim data and categorized as follows: (1) non-paclitaxel-coated devices (PBA and BMS) after entry, and (2) paclitaxel-coated devices (DEB and DES). Comorbidities, including diabetes mellitus, hypertension, and coronary heart disease, were defined from 12 months before entry, and the Charlson comorbidity index (CCI) was calculated to assess the general health status of the study participants^16^. Data on the prescription of antiplatelet agents, warfarin, and new oral anticoagulants (NOACs) were extracted using drug codes based on the Anatomical Therapeutic Chemical Classification in the claims data within the study period and were analyzed in conjunction with other information, including demographic parameters and comorbidities based on the patients’ medical records.

### Study outcomes

The primary outcome of the present study was the all-cause mortality during the follow-up period. Mortality was confirmed by using the Korea statistics database. Patients in each cohort were followed up from the index procedure until the occurrence of the primary study outcome or until December 31, 2019, whichever occurred first. This definition is intended to prevent a record of death from being missed during treatment, such as by changes in medical service providers.

The secondary outcome of our study was an amputation event after the index procedure during the follow-up period. Amputation events were defined as the procedure code of major amputation of the extremities (N0571, N0572, N0573, and N0574) from the NHIS data.

### Statistical analysis

Baseline characteristics were compared according to the groups. Categorical variables were expressed as numbers and percentages, and continuous variables were expressed as mean ± SD. Demographic characteristics of the patients presented as frequencies and percentages. The overall survival rates were evaluated using a Kaplan–Meier curve, and the log-rank test was used to compare the groups. To evaluate the effects of the prognostic variables, the Cox proportional hazards model was used for multivariate analyses, and adjustments were made for variables with statistically significant differences between the groups.

There may be a selection bias due to the differences in the proportion of basic characteristics between groups, which can be reasonably addressed by propensity score matching (PSM). The propensity score was defined as the probability of individuals with paclitaxel-coated devices being treated and was calculated with multiple logistic regression using variables that included age, sex, DM, HTN, warfarin, and NOAC. The paclitaxel-coated and non-paclitaxel-coated device groups were matched in a 1:1 proportion. Age and gender were perfectly matched between the two groups, and the other variables were matched by the Greedy nearest matching method. After PSM, Cox proportional hazards models were repeated to determine the association between usage of paclitaxel-coated devices and all-cause mortality and were reported in terms of hazard ratios (HRs) and 95% confidence intervals (CI).

SAS version 9.4 (SAS Institute Inc, Cary, NC) and R version 3.5.2 (R Foundation for Statistical Computing, Vienna, Austria) software programs were used for the analyses. Two of the authors had full access to all study data and were responsible for data integrity and data analysis accuracy.

## Results

### Characteristics of the study population

**Figure 1:**
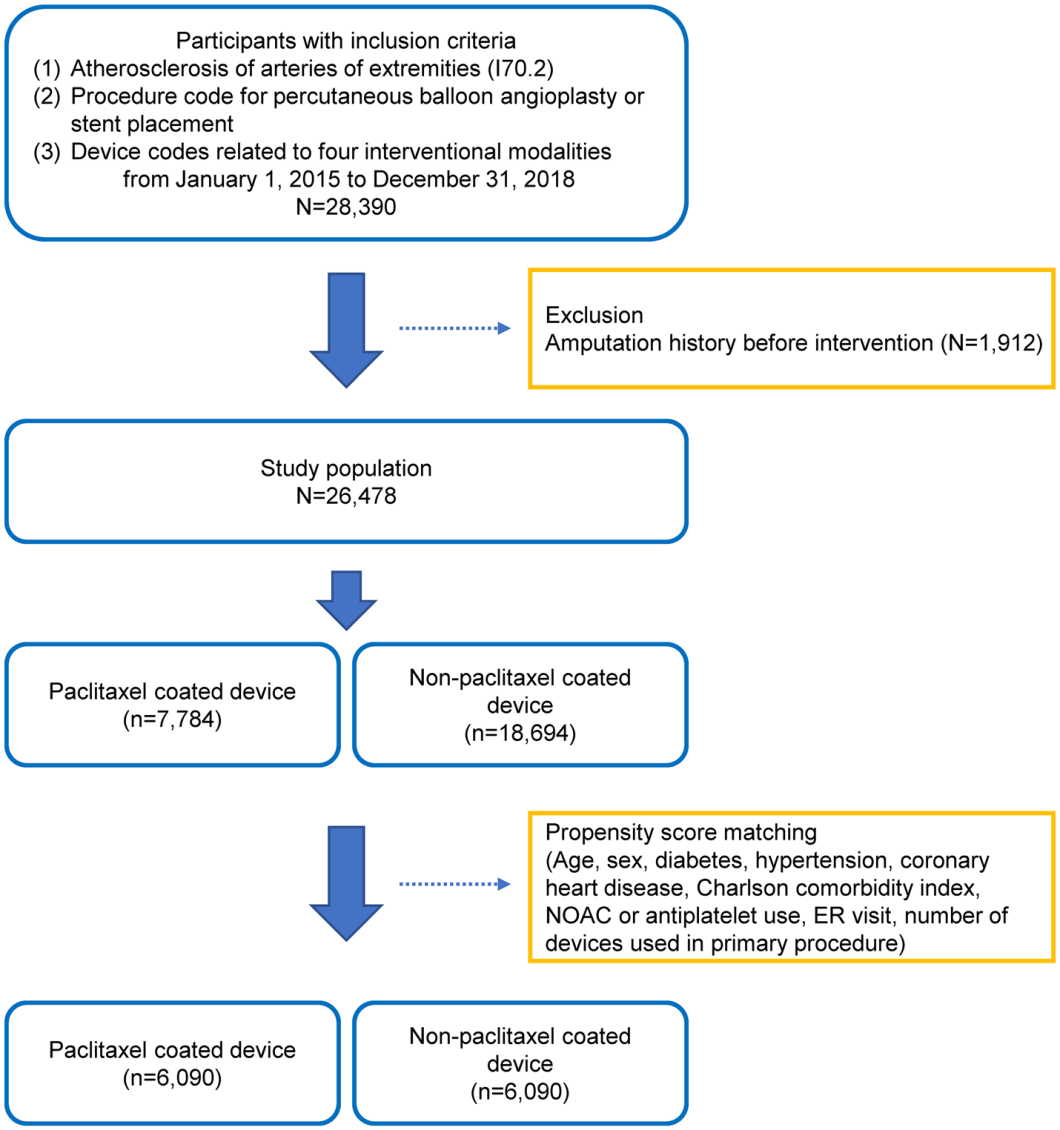
Flow chart of the study design. NOAC: new oral anticoagulants, ER: emergency room.

This study included a total of 28,390 patients with health insurance claims who had been diagnosed with atherosclerosis of native arteries in the extremities (I702) and treated with four interventional modalities (PBA, BMS, DEB, and DES) after September 2015. We excluded 1912 patients who had amputations of the lower extremities before 2015. Therefore, 26,478 patients were finally included in this study. The number of patients with a non-drug-coated device was 18,694, and the number of patients with a drug-coated device was 7784. After PSM, there were 6090 patients per cohort (Fig. 1). The baseline characteristics of the study participants are summarized in Table 1. After PSM, the mean age and sex ratio were almost the same between the two groups. Even after PSM, there were differences in comorbidities such as diabetes and hypertension and medication status such as warfarin and NOAC in the two groups. Antiplatelet usage, coronary heart disease, visits to the emergency room 3 days before the index procedure, and the number of devices used in the procedure showed no significant difference between the two groups after PSM.

**Table 1:** Baseline characteristics of study population.

	Before PSM			After PSM		
	Non-drug-coated device	Paclitaxel-coated device	*P*-value	Non-drug-coated device	Paclitaxel-coated device	*P*-value
Total	18,694	7784		6090	6090	
Age *n*	67.21 ± 11.74	68.77 ± 10.59	< 0.0001	68.86 ± 9.71	68.86 ± 9.71	1
0 ~ 9	2	0	< 0.0001	0	0	1
10 ~ 19	7	2		0	0	
20 ~ 29	84	23		5	5	
30 ~ 39	254	56		21	21	
40 ~ 49	1041	272		168	168	
50 ~ 59	3142	1023		813	813	
60 ~ 69	5550	2508		2070	2070	
70 ~ 79	6005	2757		2203	2203	
80 ~	2609	1143		810	810	
Sex *n* (%)			< 0.0001			1
Male	13,648 (73.0)	6194 (79.5)		4996 (82.0)	4996 (82.0)	
Female	5046 (27.0)	1590 (20.5)		1094 (18.0)	1094 (18.0)	
DM *n* (%)	12,673 (67.8)	5551 (71.3)	< 0.0001	4170 (68.5)	4320 (70.9)	0.0031
HTN *n* (%)	15,719 (84.1)	6608 (84.9)	0.1002	4832 (79.3)	5192 (85.3)	< 0.0001
CHD *n* (%)	8059 (43.1)	3713 (47.7)	< 0.0001	2977 (48.9)	2875 (47.2)	0.0643
Warfarin *n* (%)	1414 (7.6)	575 (7.4)	0.6187	666 (10.9)	435 (7.1)	< 0.0001
Antiplatelet *n* (%)	10,206 (54.6)	5341 (68.6)	< 0.0001	4031 (66.2)	4117 (67.6)	0.0977
NOAC *n* (%)	1625 (8.7)	716 (9.2)	0.1866	760 (12.5)	550 (9.0)	< 0.0001
Devices on procedure *n*	1.01 ± 0.11	1.03 ± 0.18	< 0.0001	1.02 ± 0.15	1.02 ± 0.14	0.2167
ER visit *n* (%)	362 (1.9)	115 (1.5)	0.0105	107 (1.8)	86 (1.4)	0.1276
CCI	2.44 ± 1.62	2.29 ± 1.56	< 0.0001	2.31 ± 1.65	2.30 ± 1.56	0.5494

Values are expressed as mean ± standard deviation (SD), or *n*(%).

*DM* diabetes mellitus, *HTN* hypertension, *CHD* coronary heart disease, *NOAC* new oral anticoagulants, *CCI* Charlson comorbidity index, *ER* emergency room.

### All-cause mortality before and after PSM

**Figure 2:**
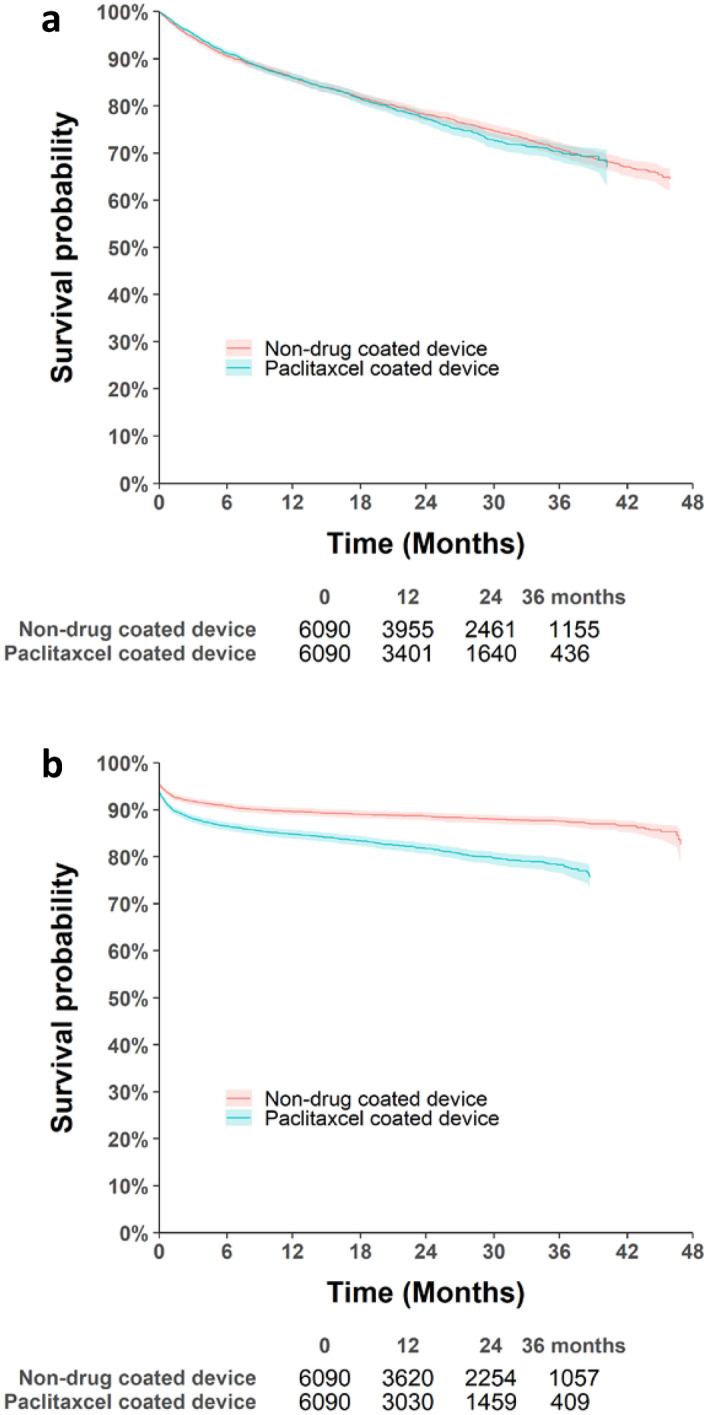
Cumulative Kaplan–Meier estimate of (**a**) all-cause mortality and (**b**) amputation-free survival in patients treated with the paclitaxel-coated and non-paclitaxel-coated devices after propensity score matching. Created by using SAS software (version 9.4; SAS Institute Inc., Cray, NC, USA).

After the index procedure, the median follow-up was 554 days (IQR 228–964 days) and 423 days (IQR 173–750 days) in the non-paclitaxel-coated device group and the paclitaxel-coated device group before PSM, respectively. After PSM, the median follow-up was 580 days (IQR 240–991 days) and 433 days (IQR 175–757 days) in the non-paclitaxel-coated device and paclitaxel-coated device groups, respectively. Deaths occurred in 3898/18,694 and 1305/6090 cases in the non-paclitaxel-coated device group and 1401/7784 and 1088/6090 cases in the paclitaxel-coated device group before and after PSM, respectively. The survival probabilities of all-cause mortality on the Kaplan–Meier curve after PSM were 86%, 78%, and 70% at 12, 14, and 36 months, respectively, in the non-paclitaxel-coated device group, and 86%, 77%, and 70% at 12, 24, and 36, in the paclitaxel-coated device group, respectively (Fig. 2a).

### Amputation before and after PSM

All events of amputation performed on lower extremities were analyzed after the index procedure until December 31, 2019. Amputation on lower extremities occurred in 1919/18694 and 670/6090 patients in the non-paclitaxel-coated device group and in 1305/7784 and 999/6090 patients in the paclitaxel-coated device group before and after PSM, respectively. The survival probabilities of amputation free survival on the Kaplan–Meier curve after PSM were 89%, 88%, and 87% at 12, 14, and 36 months, respectively, and 84%, 82%, and 78%, at 12, 24, and 36, respectively, in the non-paclitaxel-coated device group respectively (Fig. 2b).

### Univariate analysis and adjusted mortality and amputation free survival analyses

Univariate analysis and adjusted mortality analysis on all-cause mortality and amputation after PSM are presented in Tables 2 and 3. From the univariate analysis, all-cause mortality was not associated with paclitaxel-coated device use (HR 0.99; 95% CI 0.90–1.1; *P* = 0.8431). The adjusted analyses did not differ from the previous results (Table 2). After PSM, the rate of amputation event was higher in patients with a drug-coated device than a non-drug-coated device from the univariate analysis (HR 1.614; 95% CI 1.26–1.58; *P* ≤ 0.001) and the multivariable adjustment (HR 1.614; 95% CI 1.46–1.78; *P* ≥ 0.001) (Table 3).

**Table 2:** Univariate and multivariate analysis of all-cause mortality after propensity score matching.

	Reference	Crude			Adjusted		
		HR	95% CI	P-value	HR	95% CI	P-value
	Non-drug coated device	0.99	0.90–1.1	0.8431	0.992	0.91–1.08	0.8464
Sex	Male	1.311	1.19–1.45	< 0.0001	1.059	1.05–1.06	< 0.0001
Age		1.003	1–1.01	0.2371	1.1	1–1.22	0.0613
DM	No	1.273	1.16–1.4	< 0.0001	1.352	1.23–1.49	< 0.0001
HTN	No	1.291	1.15–1.44	< 0.0001	1.108	0.99–1.24	.0811
CHD	No	1.025	0.95–1.11	0.5416			
Warfarin	No	0.708	0.61–0.82	< 0.0001	0.836	0.72–0.97	0.0193
Antiplatelet	No	0.27	0.25–0.29	< 0.0001			
NOAC	No	0.52	0.44–0.61	< 0.0001	0.46	0.39–0.54	< 0.0001
CCI		1.05	1.02–1.08	0.001			
Devices on procedure	No	1.079	1.03–1.13	0.0007			
ER visit	No	1.34	1.01–1.78	0.0446			

*DM* diabetes mellitus, *HTN* hypertension, *CHD* coronary heart disease, *NOAC* new oral anticoagulants, *CCI* Charlson comorbidity index, *HR* hazard ratio, *ER* emergency room.

**Table 3:** Univariate and multivariate analysis of amputation free survival after propensity score matching.

	Reference	Crude			Adjusted		
		HR	95% CI	P-value	HR	95% CI	P-value
	Non-drug coated device	1.614	1.26–1.58	< 0.0001	1.614	1.46–1.78	< 0.0001
Sex	Male	1.329	1.18–1.49	< 0.0001	1.006	1–1.01	0.0187
Age		1.007	1–1.01	0.0061	1.275	1.13–1.43	< 0.0001
DM	No	2.498	2.19–2.85	< 0.0001	2.453	2.14–2.81	< 0.0001
HTN	No	1.19	1.04–1.36	0.0099	0.931	0.81–1.07	0.2973
CHD	No	0.736	0.67–0.81	< 0.0001			
Warfarin	No	0.724	0.6–0.87	0.0005	0.841	0.7–1.01	0.064
Antiplatelet	No	0.524	0.48–0.58	< 0.0001			
NOAC	No	0.577	0.48–0.7	< 0.0001	0.631	0.52–0.77	< 0.0001
CCI		1.098	1.07–1.13	< 0.0001			
Devices on procedure	No	1.085	1.04–1.13	.0003			
ER visit	No	2.28	1.73–3	< 0.0001			

*DM* diabetes mellitus, *HTN* hypertension, *CHD* coronary heart disease, *NOAC* new oral anticoagulants, *CCI* charlson comorbidity index, *HR* hazard ratio, *ER* emergency room.

## Discussion

We retrospectively analyzed the association between the use of paclitaxel-coated devices on lower extremities and all-cause mortality from NHIS claims data. Since the data we used did not include data at the patient level, lesion characteristics such as site, length, and severity could not be determined. Since 2015, paclitaxel-coated devices have been covered by insurance only for femo-popliteal lesions; therefore, if patients were treated with DEB and/or DES, we assumed that the PAD lesion was a femo-popliteal lesion. For the severity of disease status, we analyzed variables such as visits to the emergency room 3 days before the index procedure and the number of devices during the index procedure. Through PSM, we tried to correct for confounder imbalance between the case and control groups. Cox proportional hazards models were used to inspect the interaction between the use of paclitaxel-coated devices and mortality.

Univariate analyses showed that the use of paclitaxel-coated devices was not associated with all-cause mortality in lower extremity PAD. Even in the adjusted analyses, a persistent tendency was noted. Otherwise, the use of paclitaxel-coated devices showed lower amputation-free survival on the univariate and adjusted survival analyses. We tried to increase the sensitivity of analyses by defining non-paclitaxel-coated device group newly. We narrowed down the non-paclitaxel device group as a procedure code of only femoral arteriography, excluding iliac arteriography and tibial arteriography (Supplementary Table S2). With this, we performed the same analyses, which were all-cause mortality and amputation-free survival, in the univariate and adjusted survival analyses (Supplementary Tables S3 and S4), and observed similar results in the paclitaxel-coated device group. To exclude for confounding effects of drugs such as warfarin, NOAC, and anticoagulants, we performed two subgroup analyses: a subgroup on medication of warfarin or NOAC and a subgroup on medication of antiplatelets (Supplementary Tables S5-S10). The same analyses were performed, and similar results were observed.

Paclitaxel-coated devices have been developed to overcome notorious restenosis issues of endovascular treatments in patients with PAD. In industry-funded randomized controlled trials, DEB and DES showed superiority in patency at 1, 3, or up to 5 years compared with plain endovascular treatment in PAD^17–19^.

With the wide use of the new technology, a recent meta-analysis caused a global debate on the possible harm of the paclitaxel-coated devices. Katsanos et al. reported a significant increase in mortality rates in 2 and 5 years after angioplasty of the femoropopliteal artery with paclitaxel-coated devices (coated balloons and stents) compared to control groups without paclitaxel^8^. The most recent 2019 Global Vascular Guidelines for treating chronic limb-threatening ischemia contain an apparent statement on the uncertain risk and efficacy of these devices, weighing the need to exercise appropriate caution until data from controlled prospective studies are available^20^. Moreover, ongoing trials, such as BASIL-3 (Balloon Versus Stenting in Severe Ischemia of the Leg-3) or SWEDEPAD (Swedish Drug Elution Trial in Peripheral Arterial Disease), have been temporarily paused until this critical issue is resolved^21^.

The meta-analysis by Katsanos et al., however, had some limitations^21–23^. The included trials that were focused on and designed to address patency as a primary endpoint, i.e., they were not built up to detect mortality or clinical events. Furthermore, patient-level data were not available.

According to Food and Drug Administration's willingness to investigate the long-term safety of paclitaxel-coated devices on patient-level data, several studies have been published. Albrecht et al. performed a patient-level 2-year mortality analysis based on pooled original data from four randomized controlled trials i.e., THUNDER, FEMPAC, PACIFIER, and CONSEQUENT. Logistic regression analysis revealed that paclitaxel-coated device treatment groups were not a predictor of 2-year mortality. The paclitaxel doses per patient were not significantly different in patients that died and those who did not die during the 24-month follow-up^10^. Schneider et al. conducted a patient-level meta-analysis to examine if there is a correlation between paclitaxel exposure and mortality in patients treated with the paclitaxel drug-coated balloon (IN.PACT Admiral^Ⓡ^, Medtronic, Dublin, Ireland) in the treatment of symptomatic femoropopliteal PAD. A total of 1980 patients from two randomized controlled trials (RCTs) and two prospective studies were included. There was no significant difference in all-cause mortality between paclitaxel-coated device and non-paclitaxel-coated device within the 5 years^13^. Another patient-level industry sponsored meta-analysis showed that analyses comprising three RCTs in the paclitaxel-coated balloon (Lutonix^Ⓡ^, Bard, Murray Hill, NJ) revealed that the 5-year HR was 1.01 (95% CI, 0.68–1.52) in the aggregated LEVANT trials^11^. Recently, SWEDEPAD reported results of an unplanned interim analysis of the first 2289 enrolled patients randomly assigned to treatment with drug-coated devices or treatment with uncoated devices. During a mean follow-up of 2.49 years, there was no difference in all-cause mortality between the treatment groups among patients with chronic limb-threatening ischemia or those with intermittent claudication^24^.

The retrospective population-based studies using large-scale insurance claims were published. A nationwide, multicenter retrospective cohort study including 16,560 Centers for Medicare and Medicaid Services beneficiaries admitted for femoropopliteal artery revascularization was conducted^25^. The median follow-up was 389 days (interquartile range, 277–508 days). Among all patients' treatments, treatment with paclitaxel-coated devices was associated with a lower cumulative incidence of all-cause mortality than treatment with non-paclitaxel-coated devices within 600 days after the procedure. Another retrospective health insurance claim analysis of patients covered by the second largest insurance fund in Germany, BARMER, was performed, and results showed that paclitaxel-coated devices were associated with improved overall survival (HR 0.83; 95% CI 0.77–0.90), amputation-free survival (HR 0.85; 95% CI 0.78–0.91), and freedom from major cardiovascular events (HR 0.82; 95% CI 0.77–0.89) as compared to non-paclitaxel-coated devices at five years in CLTI^26^.

The clinical outcomes were persistently released; however, there are no obvious biological mechanisms that explain a direct link between paclitaxel and mortality. The restraint of somatic cell division reduces vascular restenosis when applied locally, and its effect is historically well illustrated in the coronary vasculature^27,28^. Peripheral paclitaxel devices use the crystalline form of the cytotoxic drug, which aids in tissue uptake and retention^29^. Despite these features, drug transfer remains inefficient, and 80–90% of paclitaxel is lost in the systemic circulation^30^. Animal studies have shown evidence of distal embolization in downstream vessels^31,32^. The half-life of paclitaxel is usually months, and the peak plasma concentration occurs soon after the procedure^33^. Any mortality linked to paclitaxel exposure would occur earlier than 2 years and beyond.

Here, we focused on the Korean population in analyzing the safety of the paclitaxel-coated device. To the best of our knowledge, this is the first study involving the whole nation. Moreover, we merged mortality data from Statistics Korea to NHIS so that almost all death events could be traceable. Like two other nationwide studies discussed earlier^25,26^, paclitaxel-coated device treatment in PAD did not increase all-cause mortality than the non-paclitaxel-coated device. Based on that, the mortality between the two groups is not different despite the disparity of co-morbidities such as diabetes and hypertension in our study, and it might suggest that the paclitaxel-coated devices did not affect the mortality of patients with PAD. Additionally, on repeated analyses with a stricter control group and two subgroups, all-cause mortality was not associated with paclitaxel-coated device use. Noticeably, our study showed that paclitaxel-coated device was a predictor of amputation of the lower extremities. The interactions between higher amputation and the use of paclitaxel-coated devices should be carefully interpreted. During endovascular procedures with DEB, up to 80% of paclitaxel does not reach the target site and is lost in the systemic circulation^34^. A hypothesis on higher amputation rate on patients treated with drug-eluting devices is downstream embolization of the paclitaxel particles^35^. Paclitaxel particles could occlude small vessels and inhibit angiogenesis at locations distant from the target site. However, further biological mechanisms should be investigated to reach a definitive conclusion.

We had several limitations in this study. Since paclitaxel-coated devices for PAD were available from 2015 in Korea and National Health Insurance Claims data were only available until 2019, the median follow-up was relatively shorter than the other studies. National Health Insurance Claims do not involve procedure-specific information; therefore, we could not subdivide data into groups such as dose-specific and lesion-specific subgroups.

In conclusion**,** with a propensity score-matched population-based retrospective analysis of paclitaxel-coated devices on PAD, no association with all-cause mortality and lower amputation-free survival was found when compared with non-paclitaxel-coated devices. Even with shortcomings, we included the whole Korean population and provided important information for physicians caring for patients with PAD.

## Supplementary Information

Supplementary Information.

## Data Availability

The datasets generated during and/or analyzed during the current study are available from the corresponding author on reasonable request.
