# Supplementary material for: Mortality is not associated with paclitaxel-coated devices usage in peripheral arterial disease of lower extremities
Source: Sci Rep. 2021 Sep 14;11:18214. doi: 10.1038/s41598-021-97675-9 (PMC8440584; doi:10.1038/s41598-021-97675-9)
Supplement: Supplementary file 1 — Supplementary Information. [file 41598_2021_97675_MOESM1_ESM.pdf]

Supplementary material

Mortality is not Associated with Paclitaxel-Coated Devices Usage in Peripheral Arterial Disease of Lower Extremities

Dai Sik Ko, MD[1*], Gi Hwan Bae, MSC[2*], Sang Tae Choi, MD, PhD[1], Jaehun Jung, MD, PhD[2,3], and Jin Mo Kang, MD, PhD[1]

[1]Division of Vascular Surgery, Department of Surgery, Gachon University Gil Medical Center, Incheon, Republic of Korea

[2]Artificial Intelligence and Big-Data Convergence Center, Gachon University Gil Medical Center, Incheon, Republic of Korea

[3]Department of Preventive Medicine, Gachon University College of Medicine, Incheon, Republic of Korea

*Dai Sik Ko and Gi Hwan Bae contributed equally to this work

Short title: Paclitaxel-coated devices and all-cause mortality

Email: Dai Sik Ko [igreg1221@gmail.com]; Gi Hwan Bae [pkhstat@gmail.com]; Sang Tae Choi [choist@gilhospital.com]

Corresponding Authors

Jin Mo Kang

Division of Vascular Surgery, Department of Surgery, Gachon University Gil Medical Center, Incheon, Republic of Korea

Address: 34 Namdong-daero 774beon-gil, Namdong-gu, Incheon 21565, Republic of Korea

Email: calzevi@gmail.com

Tel: +82-32-460-3244

Fax: +82-32-460-3247

Jaehun Jung

Department of Preventive Medicine, Gachon University College of Medicine, Incheon, Korea

Artificial Intelligence and Big-Data Convergence Center, Gachon University Gil Medical

Center, Incheon, Republic of Korea

Address: 38-13 Dokjeom, Incheon, Republic of Korea (21565)

Email: eastside1st@gmail.com

Tel: +82-32-10-6359-3201

Fax: +82-32-458-2608

Table of Contents

Supplementary Table 1. Codes for plain balloon angioplasty (PBA), bare metal stents (BMSs), drug-eluting balloons (DEBs), and drug-eluting stents (DES).

Supplementary Table 2. Baseline characteristics of study population excluding procedure codes of iliac arteriography and tibial arteriography. *DM: diabetes mellitus, HTN: hypertension, CHD: coronary heart disease, NOAC: new oral anticoagulants, CCI: charlson comorbidity index, ER: emergency room

Supplementary Table 3. Baseline characteristics of study population on medication of warfarin or NOAC. *DM: diabetes mellitus, HTN: hypertension, CHD: coronary heart disease, NOAC: new oral anticoagulants, CCI: charlson comorbidity index, ER: emergency room

Supplementary Table 4. Baseline characteristics of study population on medication of antiplatelet. *DM: diabetes mellitus, HTN: hypertension, CHD: coronary heart disease, NOAC: new oral anticoagulants, CCI: charlson comorbidity index, ER: emergency room

Supplementary Table 5. Univariate and multivariate analysis of all-cause mortality after propensity score matching of study population excluding procedure codes of iliac arteriography and tibial arteriography. *DM: diabetes mellitus, HTN: hypertension, CHD: coronary heart disease, NOAC: new oral anticoagulants, CCI: charlson comorbidity index, HR: hazard ratio, ER: emergency room

Supplementary Table 6. Univariate and multivariate analysis of amputation free survival after propensity score matching of study population excluding procedure codes of iliac arteriography and tibial arteriography. *DM: diabetes mellitus, HTN: hypertension, CHD: coronary heart disease, NOAC: new oral anticoagulants, CCI: charlson comorbidity index, HR: hazard ratio, ER: emergency room

Supplementary Table 7. Univariate and multivariate analysis of all-cause mortality after propensity score matching of study population on medication of warfarin or NOAC. *DM: diabetes mellitus, HTN: hypertension, CHD: coronary heart disease, NOAC: new oral anticoagulants, CCI: charlson comorbidity index, HR: hazard ratio, ER: emergency room

Supplementary Table 8. Univariate and multivariate analysis of amputation free survival after propensity score matching of study population on medication of warfarin or NOAC. *DM: diabetes mellitus, HTN: hypertension, CHD: coronary heart disease, NOAC: new oral anticoagulants, CCI: charlson comorbidity index, HR: hazard ratio, ER: emergency room

Supplementary Table 9. Univariate and multivariate analysis of all-cause mortality after propensity score matching of study population on medication of platelet. *DM: diabetes

mellitus, HTN: hypertension, CHD: coronary heart disease, NOAC: new oral anticoagulants, CCI: charlson comorbidity index, HR: hazard ratio, ER: emergency room

Supplementary Table 10. Univariate and multivariate analysis of amputation free survival after propensity score matching of study population on medication of platelet. *DM: diabetes mellitus, HTN: hypertension, CHD: coronary heart disease, NOAC: new oral anticoagulants, CCI: charlson comorbidity index, HR: hazard ratio, ER: emergency room

Supplementary Table 1. Codes for plain balloon angioplasty (PBA), bare metal stents (BMSs), drug-eluting balloons (DEBs), and drug-eluting stents (DES).

Class	Devices	Code
Drug coated Balloon Catheter	IN PACT ADMIRAL PACLITAXEL ELUTING BALLOON CATHETER	J4077006
	SEQUENT PLEASE OTW	J4077007
	RANGER OTW PACLITAXEL-COATED PTA BALLOON	J4077021
	LUTONIX DRUG COATED BALLOON PTA CATHETER	J4077068
	PASSEO-18 LUX DRUG ELUTING BALLOON PTA CATHETER	J4077094
Drug coated Stent	ELUVIA DRUG ELUTING VASCULAR STENT	J5238021
	ZILVER PTX DRUG ELUTING PERIPHERAL STENT-THUMBWHEEL	J5238102
PTA balloon catheter	SUB-4 SMALL VESSEL BALLOON DILATION CATHETER	J4071001
	ADMIRAL XTREME PTA BALLOON CATHETER	J4071006
	HIGH FIVE ANGIOPLASTY ULTRA-HIGH PRESSURE CATHETER	J4071009
	OPTA LP PTA BALLOON CATHETER	J4071013
	COYOTE PTA BALLOON DILATATION CATHETER	J4071020
	TRESO PTA BALLOON DILATATION CATHETER	J4071024
	SYMMETRY BALLOON DILATATION CATHETER	J4071041
	CENTRO PTA BALLOON CATHETER	J4071055
	CONQUEST PTA BALLOON CATHETER	J4071068
	NANOCROSS PTA DILATATION CATHETER	J4071073
	CRONUS II PTA BALLOON CATHETER	J4071074
	OHICHO PTA BALLOON CATHETER	J4071076
	ULTRA-SOFT SV PTA BALLOON CATHETER	J4071081
	ADVANCE 18LP LOW PROFILE PTA BALLOON DILATION CATHETER	J4071082
	STERLING MONORAIL BALLOON DILATATION CATHETER	J4071091
	INNOVATE PTA BALLOON CATHETER	J4071092
	STERLING OTW BALLOON DILATATION CATHETER	J4071101
	PACIFIC XTREME PTA BALLOON CATHETER	J4071106
	SAVVY PTA DILATATION CATHETER	J4071113
	RIVAL PTA BALLOON CATHETER	J4071168
	EVERCROSS PTA DILATATION CATHETER	J4071173
	CRONUS HIGH PRESSURE PTA BALLOON CATHETER	J4071174
	BANTAM, BANTAM FLORIAN, CLEARPAC OMEGA	J4071176
	ADVANCE 35LP LOW PROFILE PTA BALLOON DILATATION CATHETER	J4071182
	GENOSS PTA BALLOON CATHETER	J4071189
	COYOTE ES PTA BALLOON DILATATION CATHETER	J4071191
	PASSEO 35 PTA BALLOON CATHETER	J4071194
	MUSTANG BALLOON DILATATION CATHETER	J4071201
	ARMADA 14 PTA CATHETER	J4071203
	AMPHIRION DEEP BALLOON CATHETER	J4071206
	POWERFLEX EXTREME PTA BALLOON CATHETER	J4071213
	ULTRAVERSE PTA BALLOON CATHETER	J4071268
	POWERCROSS PTA DILATAION CATHETER	J4071273
	ADVANCE 14LP LOW PROFILE PTA BALLOON DILATATION CATHETER	J4071282
	STERLING SL PTA BALLOON DILATATION CATHETER	J4071291
	PASSEO	J4071294
	ARMADA 35 PTA CATHETER	J4071303
	AMPHIRION DEEP LONG CONE BALLOON CATHETER	J4071306
	CONQUEST40 PTA DILATATION CATHETER	J4071368
	PASSEO-35 HP	J4071394

	AMPHIRION DEEP BALLOON CATHETER	J4071406
	POWERFLEX PLUS PTA DILATATION CATHETER	J4071413
	SUBMARINE RAPIDO PTA BALLOON CATHETER	J4071506
	POWERFLEX P3 PTA BALLOON DILATATION CATHETER	J4071513
	REEF HP PTA BALLOON CATHETER	J4071606
	OPTA PRO PTA LOW-PROFILE BALLOON DILATATION CATHETER	J4071613
	AMPHIRION DEEP RAPID EXCHANGE(RX) BALLOON CATHETER	J4071706
	AMIIA RAPID EXCHANGE BALLOON CATHETER	J4071713
	AMPHIRION DEEP RAPID EXCHANGE(RX) LONG CONE BALLOON CATHETER	J4071806
	FORTREX PTA BALLOON CATHETER	J4071906
	AVIATOR PLUS PTA BALLOON DILATATION CATHETER	J4071913
	SLEEK PTA BALLOON CATHETER	J4071923
	SAVVY LONG PTA BALLOON DILATATION CATHETER	J4071933
	SLEEK OTW PTA BALLOON CATHETER	J4071943
	POWERFLEX PRO PTA BALLOON CATHETER	J4071953
	ADMIRAL XTREME PTA BALLOON CATHETER	J4072006
	ULTRA THIN BALLOON DILATATION CATHETER	J4072011
	POWER FLEX PLUS PTA BALLOON CATHETER	J4072013
	ULTRA THIN DIAMOND PTA BALLOON CATHETER	J4072031
	BLUE-MAX BALLOON DILATATION	J4072051
	CONQUEST PTA BALLOON CATHETER	J4072068
	EVERCROSS PTA DILATATION CATHETER	J4072073
	CRONUS II PTA BALLOON CATHETER	J4072074
	OHICHO PTA BALLOON CATHETER	J4072076
	ADVANCE 18LP LOW PROFILE PTA BALLOON DILATION CATHETER	J4072082
	INNOVATE PTA BALLOON CATHETER	J4072092
	ARMADA 35 PTA CATHETER	J4072103
	PACIFIC XTREME PTA BALLOON CATHETER	J4072106
	OPTA LP PTA BALLOON CATHETER	J4072113
	ATLAS PTA BALLOON DILATATION CATHETER	J4072168
	POWERCROSS PTA DILATAION CATHETER	J4072173
	CRONUS HIGH PRESSURE PTA BALLOON CATHETER	J4072174
	BANTAM, BANTAM FLORIAN, CLEARPAC OMEGA	J4072176
	ADVANCE 35LP LOW PROFILE PTA BALLOON DILATATION CATHETER	J4072182
	PASSEO 35 PTA BALLOON CATHETER	J4072194
	SUBMARINE RAPIDO PTA BALLOON CATHETER	J4072206
	POWERFLEX EXTREME PTA BALLOON CATHETER	J4072213
	RIVAL PTA BALLOON CATHETER	J4072268
	PASSEO	J4072294
	REEF HP PTA BALLOON CATHETER	J4072306
	ULTRAVERSE PTA BALLOON CATHETER	J4072368
	SAVVY PTA DILATATION CATHETER	J4072413
	ULTRAVERSE 035 PTA DILATATION CATHETER	J4072468
	POWERFLEX P3 PTA BALLOON DILATATION CATHETER	J4072513
	OPTA PRO PTA LOW-PROFILE BALLOON DILATATION CATHETER	J4072613
	AMIIA RAPID EXCHANGE BALLOON CATHETER	J4072713
	AVIATOR PLUS PTA BALLOON DILATATION CATHETER	J4072913
	SAVVY LONG PTA BALLOON DILATATION CATHETER	J4072923
	POWERFLEX PRO PTA BALLOON CATHETER	J4072933
	ARMADA 35 PTA CATHETER	J4073003
	XXL BALLOON DILATATION CATHETER	J4073011

	MAXI LD PTA BALLOON CATHETER	J4073013
	ATLAS PTA BALLOON DILATATION CATHETER	J4073068
	PACIFIC PLUS PTA BALLOON CATHETER	J8071073
	SABER PTA DILATION CATHETER	J8071963
	ARMADA14XT PTA CATHETER	J8072040
	RAPIDCROSS	J8072073
	ARMADA14XT PTA CATHETER	J8072140
	ATLAS GOLD PTA DILATATION CATHETER	J8073068
	ALL-TERRAIN BALLOON DILATATION CATHETER	J4075102
	SYNERGY BALLOON DILATATION CATHETER	J4076001
	MUSTANG BALLOON DILATATION CATHETER	J4076011
	ALL-TERRAIN BALLOON DILATATION CATHETER	J4076102
	GENOSS PTA BALLOON CATHETER	J8071289
Bare Metal Stent	CHROMIS DEEP STENT	J5233016
	NITI-S STENT	J5233019
	CAROTID WALL STENT	J5233021
	EXPANDER STENT	J5233026
	MISAGO RX SELF EXPANDING STENT SYSTEM	J5233029
	WALLSTENT ENDOPROSTHESIS WITH UNISTEP PLUS	J5233031
	ZILVER VASCULAR STENT	J5233032
	RX ACCULINK CAROTID STENT SYSTEM	J5233040
	EXPRESS VASCULAR LD PREMOUNTED STENT SYSTEM	J5233061
	EXPRESS SD MONORAIL PREMOUNTED STENT SYSTEM	J5233064
	LUMINEXX3 VASCULAR STENT	J5233068
	EPIC NITINOL VASCULAR STENT SYSTEM	J5233071
	PROTEGE RX CAROTID STENT	J5233073
	INNOVA SELF-EXPANDING STENT SYSTEM	J5233081
	ZILVER FLEX VASCULAR STENT	J5233082
	GENOSS PERIPHERAL STENT SYSTEM	J5233089
	HERCULES VASCULAR	J5233090
	DYNAMIC PERIPHERAL STENT SYSTEM	J5233094
	COMPLETE SE	J5233106
	LIFESTENT	J5233168
	PROTEGE EVERFLEX SELF-EXPANDING PERIPHERAL STENT	J5233173
	PULSAR	J5233194
	CRISTALLO IDEALE STENT	J5233206
	SMART CONTROL NITINOL STENT	J5233213
	OMNILINK ELITE PERIPHERAL STENT SYSTEM	J5233240
	LIFESTREAM	J5233268
	VISI-PRO OTW BALLOON EXPANDABLE STENTS	J5233273
	ASTRON	J5233294
	HIPPOCAMPUS RENAL STENT	J5233306
	PALMAZ GENESIS PERIPHERAL STENT	J5233313
	RX HERCULINK ELITE PERIPHERAL STENT SYSTEM	J5233340
	PARAMOUNT MINI GPS STENT AND DELIVERY SYSTEM	J5233373
	MARIS DEEP STENT	J5233406
	PRECISE RX NITINOL STENT	J5233413
	ABSOLUTE PRO LL PERIPHERAL SELF-EXPANDING STENT SYSTEM	J5233440
	PALMAZ BLUE .014	J5233513
	ABSOLUTE PRO PERIPHERAL SELF-EXPANDING STENT SYSTEM	J5233540
	PROTEGE GPS SEIF-EXPANDING PERIPHERAL STENT	J5233573
	ASSURANT STENT	J5233606
	PALMAZ BLUE .018	J5233613
	SMARTFLEX VASCULAR STENT SYSTEM	J5233713

	SUPERA PERIPHERAL STENT SYSTEM	J8233040
	EVERFLEX SELF-EXPANDING PERIPHERAL STENT	J8233473

Supplementary Table 2. Baseline characteristics of study population excluding procedure codes of iliac arteriography and tibial arteriography. *DM: diabetes mellitus, HTN: hypertension, CHD: coronary heart disease, NOAC: new oral anticoagulants, CCI: charlson comorbidity index, ER: emergency room

	Before PSM			After PSM		
	Non-drug coated device	Paclitaxel coated device	*P*-value	Non-drug coated device	Paclitaxel coated device	*P*-value
Total	2443	7784		2148	2148	
Age *n*	69.29±10.61	68.77±10.59	.0356	69.15±9.39	69.15±9.39	1
10~19	0	2	.0776	0	0	1
20~29	2	23		0	0	
30~39	15	56		3	3	
40~49	88	272		56	56	
50~59	331	1023		297	297	
60~69	731	2508		683	683	
70~79	868	2757		817	817	
80~	408	1143		292	292	
Sex *n* (%)						
Male	1940(79.4)	6194(79.6)	.8618	1783(83)	1783(83)	1
Female	503(20.6)	1590(20.4)		365(17)	365(17)	
DM *n* (%)	1581(64.7)	5551(71.3)	<.0001	1473(68.6)	1451(67.6)	.4716
HTN *n* (%)	2054(84.1)	6608(84.9)	.3289	1838(85.6)	1840(85.7)	.9307
CHD *n* (%)	1175(48.1)	3713(47.7)	.7323	1064(49.5)	1047(48.7)	.6039
Warfarin *n* (%)	169(6.9)	575(7.4)	.436	145(6.8)	155(7.2)	.5494
Antiplatelet *n* (%)	1595(65.3)	5341(68.6)	.0021	1440(67)	1472(68.5)	.2961
NOAC*n* (%)	228(9.3)	716(9.2)	.8413	192(8.9)	172(8)	.2732
Devices on procedure *n*	1.01±0.12	1.03±0.18	<.0001	1.01±0.12	1.01±0.10	.1213
ER visit *n* (%)	47(1.9)	115(1.5)	.1231	39(1.8)	21(1)	.0193
CCI	2.19±1.57	2.29±1.56	.0081	2.24±1.56	2.27±1.54	.5883

Values are expressed as mean ± standard deviation (SD), or *n*(%)

Supplementary Table 3. Baseline characteristics of study population on medication of warfarin or NOAC. *DM: diabetes mellitus, HTN: hypertension, CHD: coronary heart disease, NOAC: new oral anticoagulants, CCI: charlson comorbidity index, ER: emergency room

	Before PSM			After PSM		
	Non-drug coated device	Paclitaxel coated device	*P*-value	Non-drug coated device	Paclitaxel coated device	*P*-value
Total	361	1195		263	263	
Age *n*	70.53±10.60	68.29±11.58	.7274	70.21±9.18	70.21±9.18	1
10~19	0	1	<.0001	0	0	1
20~29	0	10		1	1	
30~39	3	18		5	5	
40~49	10	49		29	29	
50~59	40	145		75	75	
60~69	97	353		113	113	
70~79	134	457		40	40	
80~	77	162				
Sex *n* (%)						
Male	278(77)	956(80)	.8082	231(87.8)	231(87.8)	1
Female	83(23)	239(20)		32(12.2)	32(12.2)	
DM *n* (%)	214(59.3)	742(62.1)	<.0001	164(62.4)	155(58.9)	.4218
HTN *n* (%)	306(84.8)	993(83.1)	.1485	224(85.2)	217(82.5)	.407
CHD *n* (%)	168(46.5)	562(47)	.4505	120(45.6)	121(46)	.9303
Warfarin *n* (%)	169(46.8)	575(48.1)		123(46.8)	122(46.4)	.9303
Antiplatelet *n* (%)	241(66.8)	864(72.3)	.0034	190(72.2)	190(72.2)	1
NOAC *n* (%)	228(63.2)	716(59.9)		164(62.4)	161(61.2)	.7878
Devices on procedure *n*	1.02±0.16	1.03±0.18	.528	1.02±0.14	1.02±0.15	.7611
ER visit *n* (%)	8(2.2)	22(1.8)	.2379	7(2.7)	1(0.4)	.0325
CCI	2.07±1.45	2.28±1.54	.2808	2.16±1.43	2.09±1.35	.5727

Values are expressed as mean ± standard deviation (SD), or *n*(%)

Supplementary Table 4. Baseline characteristics of study population on medication of antiplatelet. *DM: diabetes mellitus, HTN: hypertension, CHD: coronary heart disease, NOAC: new oral anticoagulants, CCI: charlson comorbidity index, ER: emergency room

	Before PSM			After PSM		
	Non-drug coated device	Paclitaxel coated device	*P*-value	Non-drug coated device	Paclitaxel coated device	*P*-value
Total	1595	5341		1413	1413	
Age *n*	68.64±10.22	68.29±1030	.2339	68.71±9.12	68.71±912	1
10~19	0	1	.4614	0	0	1
20~29	1	17		0	0	
30~39	9	38		3	3	
40~49	56	180		34	34	
50~59	231	747		200	200	
60~69	511	1787		482	482	
70~79	564	1899		527	527	
80~	223	672		167	167	
Sex *n* (%)						
Male	1299(81.4)	4392(82.2)	.4708	1204(85.2)	1204(85.2)	1
Female	296(18.6)	949(17.8)		209(14.8)	209(14.8)	
DM *n* (%)	996(62.4)	3752(70.2)	<.0001	933(66)	940(66.5)	.7806
HTN *n* (%)	1335(83.7)	4513(84.5)	.4418	1200(84.9)	1212(85.8)	.5232
CHD *n* (%)	731(45.8)	2535(47.5)	.2518	665(47.1)	696(49.3)	.2432
Warfarin *n* (%)	119(7.5)	416(7.8)	.6666	104(7.4)	102(7.2)	.8849
Antiplatelet *n* (%)	1595(100)	5341(100)		1413(100)	1413(100)	
NOAC *n* (%)	145(9.1)	515(9.6)	.5101	123(8.7)	118(8.4)	.7363
Devices on procedure *n*	1.01±0.12	1.03±0.18	<.0001	1.01±0.12	1.01±0.12	1
ER visit *n* (%)	24(1.5)	71(1.3)	.597	21(1.5)	12(0.8)	.115
CCI	2.04±1.47	2.16±1.50	.0045	2.07±1.43	2.11±1.41	.4359

Values are expressed as mean ± standard deviation (SD), or *n*(%)

Supplementary Table 5. Univariate and multivariate analysis of all-cause mortality after propensity score matching of study population excluding procedure codes of iliac arteriography and tibial arteriography. *DM: diabetes mellitus, HTN: hypertension, CHD: coronary heart disease, NOAC: new oral anticoagulants, CCI: charlson comorbidity index, HR: hazard ratio, ER: emergency room

		Crude			Adjusted		
	Reference	HR	95%CI	P-value	HR	95% CI	P-value
	Non-drug coated device	0.985	0.89-1.09	.7689	0.92	0.80-1.06	.2382
Sex	Male	1.39	1.26-1.54	<.0001	1.274	1.08-1.51	.0048
Age		1.057	1.05-1.06	<.0001	1.06	1.05-1.07	<.0001
DM	No	1.214	1.10-1.34	.0001	1.307	1.12-1.52	.0006
HTN	No	1.366	1.19-1.57	<.0001	1.293	1.05-1.60	.0168
CHD	No	1.071	0.98-1.17	.1276	1.169	1.02-1.34	.0244
Warfarin	No	0.584	0.48-0.71	<.0001	0.686	0.51-0.92	.0112
Antiplatelet	No	0.259	0.24-0.28	<.0001	0.273	0.24-0.31	<.0001
NOAC	No	0.597	0.50-0.72	<.0001	0.623	0.47-0.83	.0013
CCI		1.241	1.21-1.27	<.0001	1.283	1.23-1.33	<.0001
Devices on procedure	No	1.026	0.78-1.34	.8533	1.656	0.99-2.76	.053
ER visit	No	1.491	1.1-2.02	.0095	1.073	0.61-1.9	.8093

Supplementary Table 6. Univariate and multivariate analysis of amputation free survival after propensity score matching of study population excluding procedure codes of iliac arteriography and tibial arteriography. *DM: diabetes mellitus, HTN: hypertension, CHD: coronary heart disease, NOAC: new oral anticoagulants, CCI: charlson comorbidity index, HR: hazard ratio, ER: emergency room

		Crude			Adjusted		
	Reference	HR	95%CI	P-value	HR	95% CI	P-value
	Non-drug coated device	2.246	1.94-2.6	<.0001	1.828	1.53-2.19	<.0001
Sex	Male	1.5	1.34-1.68	<.0001	1.6	1.31-1.96	<.0001
Age		0.998	0.99-1	.453	0.999	0.99-1.01	.7564
DM	No	2.637	2.29-3.04	<.0001	2.466	1.95-3.12	<.0001
HTN	No	1.173	1.01-1.36	.033	1.102	0.85-1.42	.4542
CHD	No	0.786	0.71-0.87	<.0001	0.732	0.61-0.87	.0006
Warfarin	No	0.744	0.60-0.92	.006	0.779	0.54-1.13	.1851
Antiplatelet	No	0.486	0.44-0.54	<.0001	0.416	0.35-0.50	<.0001
NOAC	No	0.551	0.44-0.69	<.0001	0.671	0.47-0.97	.0312
CCI		1.085	1.05-1.12	<.0001	1.122	1.06-1.18	<.0001
Devices on procedure	No	1.399	1.08-1.81	.011	0.837	0.35-2.02	.6919
ER visit	No	2.322	1.75-3.09	<.0001	2.448	1.47-4.09	.0006

Supplementary Table 7. Univariate and multivariate analysis of all-cause mortality after propensity score matching of study population on medication of warfarin or NOAC. *DM: diabetes mellitus, HTN: hypertension, CHD: coronary heart disease, NOAC: new oral anticoagulants, CCI: charlson comorbidity index, HR: hazard ratio, ER: emergency room

		Crude			Adjusted		
	Reference	HR	95%CI	P-value	HR	95% CI	P-value
	Non-drug coated device	1.049	0.77-1.43	.7619	1.195	0.75-1.92	.4576
Sex	Male	1.008	0.72-1.42	.9632	1.187	0.61-2.32	.6142
Age		1.072	1.06-1.09	<.0001	1.082	1.05-1.12	<.0001
DM	No	1.114	0.84-1.49	.4604	1.001	0.62-1.61	.9973
HTN	No	1.825	1.15-2.9	.0106	3.163	1.15-8.67	.0252
CHD	No	0.982	0.75-1.29	.8981	1.063	0.67-1.69	.7965
Warfarin	No	0.856	0.65-1.13	.2686	0.944	0.59-1.5	.8076
Antiplatelet	No	0.612	0.46-0.82	.0008	0.74	0.45-1.22	.2393
NOAC	No	0.999	0.76-1.32	.9961	0.898	0.56-1.43	.653
CCI		1.184	1.09-1.29	<.0001	1.171	1-1.37	.0461
Devices on procedure	No	1.014	0.45-2.28	.974	1.341	0.33-5.47	.6828
ER visit	No	1.151	0.43-3.1	.7811	0.754	0.11-5.43	.7796

Supplementary Table 8. Univariate and multivariate analysis of amputation free survival after propensity score matching of study population on medication of warfarin or NOAC. *DM: diabetes mellitus, HTN: hypertension, CHD: coronary heart disease, NOAC: new oral anticoagulants, CCI: charlson comorbidity index, HR: hazard ratio, ER: emergency room

		Crude			Adjusted		
	Reference	HR	95%CI	P-value	HR	95% CI	P-value
	Non-drug coated device	2.398	1.52-3.79	.0002	1.413	0.72-2.76	.312
Sex	Male	1.356	0.96-1.93	.0889	0.658	0.2-2.15	.4889
Age		1	0.99-1.01	1	1.003	0.97-1.04	.8711
DM	No	2.563	1.75-3.76	<.0001	5.206	1.84-14.75	.0019
HTN	No	0.962	0.64-1.45	.8517	1.164	0.45-3	.7537
CHD	No	0.867	0.64-1.18	.3651	0.675	0.34-1.34	.2604
Warfarin	No	1.246	0.92-1.7	.1624	0.938	0.48-1.83	.8502
Antiplatelet	No	0.71	0.51-0.98	.0379	0.471	0.24-0.92	.0276
NOAC	No	0.713	0.53-0.97	.0307	0.823	0.42-1.61	.5687
CCI		1.123	1.02-1.23	.0144	1.103	0.88-1.39	.4002
Devices on procedure	No	1.209	0.54-2.73	.6485	-	-	-
ER visit	No	0.997	0.32-3.13	.9961	-	-	-

Supplementary Table 9. Univariate and multivariate analysis of all-cause mortality after propensity score matching of study population on medication of platelet. *DM: diabetes mellitus, HTN: hypertension, CHD: coronary heart disease, NOAC: new oral anticoagulants, CCI: charlson comorbidity index, HR: hazard ratio, ER: emergency room

		Crude			Adjusted		
	Reference	HR	95%CI	P-value	HR	95% CI	P-value
	Non-drug coated device	0.963	0.83-1.12	.6267	0.879	0.71-1.08	.2291
Sex	Male	1.185	1-1.4	.0498	1.215	0.93-1.58	.1462
Age		1.058	1.05-1.07	<.0001	1.061	1.05-1.08	<.0001
DM	No	1.178	1.01-1.37	.0331	1.267	1.01-1.58	.0374
HTN	No	1.356	1.1-1.67	.0043	1.272	0.93-1.74	.1302
CHD	No	1.031	0.9-1.18	.6606	1.156	0.95-1.42	.1591
Warfarin	No	0.896	0.7-1.15	.3894	1.134	0.79-1.62	.4895
Antiplatelet	No	-	-	-	-	-	-
NOAC	No	0.937	0.74-1.19	.5954	0.905	0.62-1.31	.5992
CCI		1.208	1.16-1.26	<.0001	1.245	1.17-1.33	<.0001
Devices on procedure	No	0.791	0.49-1.28	.3371	1.02	0.42-2.47	.9652
ER visit	No	1.769	1.12-2.79	.014	1.245	1.17-1.33	<.0001

Supplementary Table 10. Univariate and multivariate analysis of amputation free survival after propensity score matching of study population on medication of platelet. *DM: diabetes mellitus, HTN: hypertension, CHD: coronary heart disease, NOAC: new oral anticoagulants, CCI: charlson comorbidity index, HR: hazard ratio, ER: emergency room

		Crude			Adjusted		
	Reference	HR	95%CI	P-value	HR	95% CI	P-value
	Non-drug coated device	2.719	2.19-3.37	<.0001	2.23	1.72-2.9	<.0001
Sex	Male	1.465	1.25-1.72	<.0001	1.376	1.01-1.87	.0425
Age		0.999	0.99-1.01	.6708	1.005	0.99-1.02	.4531
DM	No	2.768	2.29-3.35	<.0001	3.046	2.16-4.3	<.0001
HTN	No	1.194	0.98-1.46	.0796	1.311	0.9-1.92	.1629
CHD	No	0.746	0.65-0.86	<.0001	0.688	0.54-0.88	.0033
Warfarin	No	0.975	0.76-1.25	.8446	0.816	0.49-1.35	.4297
Antiplatelet	No	-	-	-	-	-	-
NOAC	No	0.592	0.44-0.79	.0004	0.735	0.45-1.2	.219
CCI		2.949	2.04-4.27	<.0001	1.084	1-1.18	.0523
Devices on procedure	No	1.39	0.98-1.97	.0659	1.164	0.43-3.13	.7626
ER visit	No	2.949	2.04-4.27	<.0001	3.892	2.07-7.33	<.0001
